# T1 difficulty does not modulate the magnitude of the attentional blink

**DOI:** 10.1177/17470218211054750

**Published:** 2021-10-26

**Authors:** Thomas M Spalek, Hayley E P Lagroix, Vincent Di Lollo

**Affiliations:** Department of Psychology, Simon Fraser University, Burnaby, BC, Canada

**Keywords:** Attention, attentional blink, T1 difficulty, response ceiling, accuracy, reaction time

## Abstract

When the visual system is busy processing one stimulus, it has problems processing a subsequent stimulus if it arrives soon after the first. Laboratory studies of this second-stimulus impairment—known as *attentional blink* (*AB*)—have employed two targets (T1, T2) presented in rapid sequence, and have found identification accuracy to be nearly perfect for T1, but impaired for T2. It is commonly believed that the magnitude of the AB is related directly to the difficulty of T1: the greater the T1 difficulty, the larger the AB. A survey of the experimental literature disconfirms that belief showing it to have arisen from artificial constraints imposed by the 100% limit of the response scale. Removal of that constraint, either using reaction time (RT) instead of accuracy as the dependent measure, or in experiments in which the functions of T2 accuracy over lags do not converge to the limit of the response scale, reveals parallel functions for the easy-T1 and the hard-T1 conditions, consistent with the idea that T1 difficulty does not modulate AB magnitude. This finding is problematic for all, but the Boost and Bounce (B&B) and the Locus Coeruleus–Norepinephrine (LC–NE) theories in which T1 acts merely as a trigger for an eventual refractory period that leads to the failure to process T2, rendering T1 difficulty and its relationship to the AB an irrelevant consideration.

Limitations in visual information processing are revealed by a phenomenon known as the *attentional blink* (AB). When two targets (T1, T2) are inserted in a stream of distractors displayed in rapid serial visual presentation (RSVP), identification accuracy is nearly perfect for T1 but is substantially reduced for T2 (Raymond et al., 1992). To study the temporal course of the AB, the lag between the two targets is varied systematically in steps of about 100 ms. The AB occurs not only in the laboratory but also in everyday life. For example, suppose that you are driving in city traffic and a kid playing on the side of the road kicks a ball onto the road. The automatic deployment of attention to the ball may impair redeployment of attention to the car in front of you suddenly applying the brakes.

Several theories have ascribed the AB to a delay in processing T2 while the system is busy with T1. During the delay, the T2 representation is said to be degraded over time and to be masked by ensuing items in the RSVP stream. For example, the attentional dwell-time hypothesis (Ward et al., 1996) holds that the AB occurs because resources required in common by the two targets are unavailable for T2 while T1 is being processed. A similar idea underlies the two-stage model of Chun and Potter (1995) in which the AB is said to occur when T2 arrives while high-level processing resources are preempted by T1. Jolicœur and Dell’Acqua’s (1998) psychological refractory period (PRP) model is similarly predicated on resource limitations leading to a bottleneck at a late stage of processing. This is also the case for the interference model of Shapiro et al. (1994) in which resources are said to be allocated in large part to the first target, and in diminishing amounts to ensuing items. A similar concept is at the basis of the model proposed by Nieuwenstein et al. (2005).

A major implication of these models is that the magnitude of the AB should be a function of the difficulty of processing T1. This is because a difficult T1 would preempt a greater proportion of resources, causing the processing of the relatively unattended T2 to be more delayed, thus extending the period for which it remains vulnerable to masking by ensuing items. A correspondingly larger T2 deficit would then follow. In everyday viewing, this is equivalent to dividing attention to two rapidly successive visual events. Deployment of attention to the second event will occur more slowly if the first event is more attentionally demanding.

The principal objective of the present work was to assess the empirical support for the proposition that T1 difficulty modulates the magnitude of the AB. To anticipate, the bulk of the evidence strongly suggests that T1 difficulty modulates the overall level of T2 performance, but not the magnitude of the AB.

Comprehensive reviews of the AB literature have been reported by Dux and Marois (2007) and by Martens and Wyble (2010). MacLean and Arnell (2012) have presented a detailed and innovative review of AB methodologies. The present approach is intended to be selective rather than comprehensive. Studies were selected that were most pertinent to—and best illustrated—the specific issue under discussion.

## Methodological considerations

Before delving into the relationship between T1 difficulty and AB magnitude, it is necessary to consider some methodological issues regarding the type of measure used to estimate the AB. This is a necessary first step because the literature contains numerous instances of faulty estimates arising from inappropriate methodology. Many of these issues have been considered in MacLean and Arnell’s (2012) examination of AB methodology. The present work focusses on how such methodological issues may affect an assessment of the relationship between T1 difficulty and AB magnitude.

Most studies of that relationship have employed identification accuracy of T2 as the dependent measure. The magnitude of the AB has been measured in one of two ways. One was to estimate the slope of the function of T2 accuracy over T1–T2 lags. A measure homologous to the slope has often been used by estimating the difference between the maximum level of T2 accuracy (MAX; usually at one of the longer lags) and the minimum level of T2 accuracy (MIN; usually at one of the shorter lags). The larger the MAX-MIN difference the larger the estimated AB (e.g., Arnell et al., 2006). Obviously, the MAX-MIN method assumes a generally progressive improvement in T2 performance as the T1–T2 lag is increased. This is invariably the case for lags beyond Lag 1. At Lag 1, the phenomenon of Lag-1 sparing may obfuscate both the slope and the MAX-MIN measures. Other studies have estimated the magnitude of the AB by measuring the area between the function of T2 accuracy over lags and either (a) a corresponding control function (e.g., Hari et al., 1999) or (b) the limit of the 100% response scale (e.g., Shapiro et al., 1994): the larger the area, the larger the estimated size of the AB.

**Figure 1.**
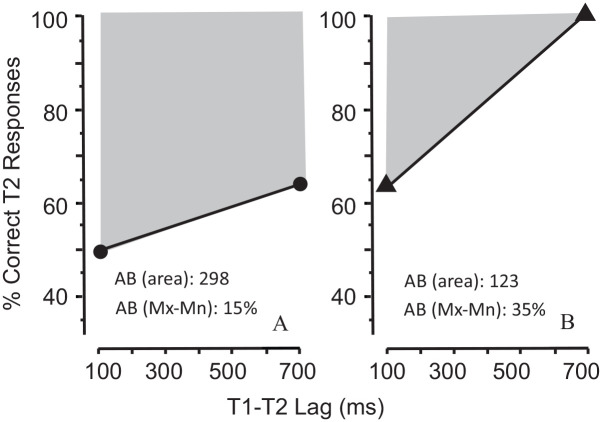
Area versus slope measures of the AB. *Note*. Comparison of two methods of estimating AB magnitude in two imaginary experiments (A and B). The *area* method (shaded regions) estimates the area of the region bounded by the function of T2 accuracy over lags and the 100% ceiling of the response scale. The *slope* estimate is obtained by subtracting the lowest level of T2 accuracy (Mn) from the corresponding highest level (Mx). The two methods yield inconsistent estimates across the two experiments. The area measure shows the AB to be greater in Experiment A; the slope measure shows the opposite.

Estimates obtained with the area and the MAX-MIN methods have been regarded as equivalent. But, as pointedly noted by MacLean and Arnell (2012), the two methods yield estimates that are far from equivalent. Consider the results of two hypothetical experiments (A and B) illustrated in Figure 1. The objective is to estimate the magnitude of the AB. On the assumption of equivalence, the “area” and the “MAX-MIN” methods should yield comparable estimates. But they do not. Figure 1 shows that the area measure yields a greater AB in Experiment A than in Experiment B (298 vs 123). In contrast, the MAX-MIN measure indicates the reverse: the AB is greater in Experiment B than in Experiment A. Clearly, the two measures cannot both be regarded as equivalently valid estimates of the AB.

**Figure 2.**
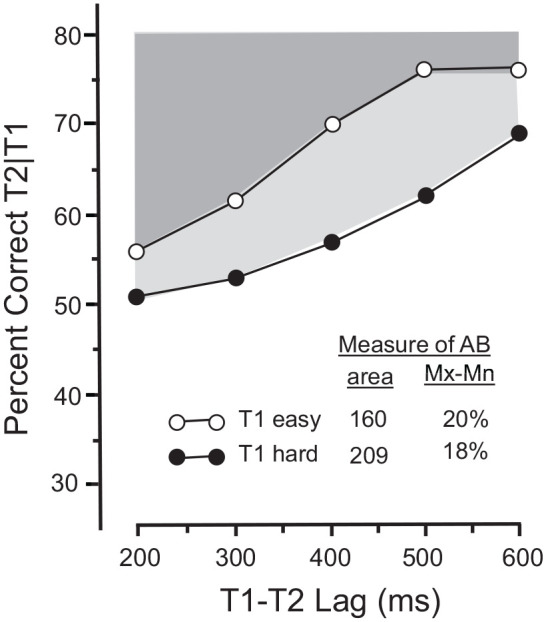
Area and slope measures yield inconsistent measures of the AB. *Source.* Adapted from Chun and Potter (1995, Figure 5). *Note*. The shaded regions represent AB magnitudes estimated by the area method. Shown in light grey is the area estimate of the AB in the hard-T1 condition. The estimate for the easy–T1 condition is shown in dark grey which occludes the upper portion of the light grey region. As was the case in Figure 1, the area and slope measures yield inconsistent estimates of AB magnitude. The area measure reveals a larger AB when T1 is hard; the slope (Mx-Mn) measure shows a slight reversal from the area estimate.

Similar contradictory outcomes can be found in actual experiments. For example, Chun and Potter (1995, Experiment 4) concluded that the magnitude of the AB was greater when T1 was masked by a digit (hard T1) than when it was masked by a keyboard symbol (easy T1). That conclusion, however, was based on the *level* of the two functions (equivalent to the area measure), as distinct from their slopes. Chun and Potter’s Figure 6A has been redrawn in Figure 2 to illustrate the inconsistency between the area and the slope estimates. The two methods yielded very different estimates. The area measure yielded a greater AB when T1 was hard than when it was easy; that difference vanished, however, for the MAX-MIN estimates which, if anything, revealed the opposite order. As noted above, the two measures cannot both be regarded as equivalently valid estimates of the AB.

Comparable inconsistencies were revealed in a study by Seiffert and Di Lollo (1997). In Experiment 1, the difficulty of T1 was manipulated by varying the contents of the RSVP frame directly following T1. That frame was either blank (easy T1) or it contained a digit that acted as a backward mask (hard T1). From the functions in their Figure 1A, we estimated the magnitude of the AB using both the area and the MAX-MIN methods. The area estimates revealed a greater AB when T1 was hard (243) than when it as easy (157). In contrast, the MAX-MIN estimates revealed the reverse: the AB was greater when T1 was easy (14%) than when it was hard (9%). A corresponding inconsistency was in evidence in their Experiment 3 in which T1 difficulty was manipulated by the number of letters in the T1 frame: T1 was either one letter (easy T1) or two letters (hard T1). The area estimate revealed a larger AB when T1 was hard (156) than when it was easy (144) but, as was the case in Experiment 1, the MAX-MIN estimate revealed the reverse: the AB was larger when T1 was easy (32%) than when it was hard (23%). Again, the two measures cannot both be regarded as providing equivalent estimates of the AB.

### Distinguishing between the area (level) and the MAX-MIN (slope) methods

In light of these inconsistencies, it seems appropriate to look for ways of distinguishing between the two methods, and to ask what criteria can be adopted for deciding between them. A first step is to acknowledge that the AB is an effect that occurs across lags. The criticality of this dependency on lag has been emphasised by MacLean and Arnell (2012) in their treatise on AB methodology. In the section entitled “The function’s slope defines the AB, not the height,” they noted that “The primary criterion that defines the AB is a lag-dependent effect of T2 performance” (p. 1087). This clearly points to the MAX-MIN (slope) measure as the appropriate index of AB magnitude.

**Figure 3.**
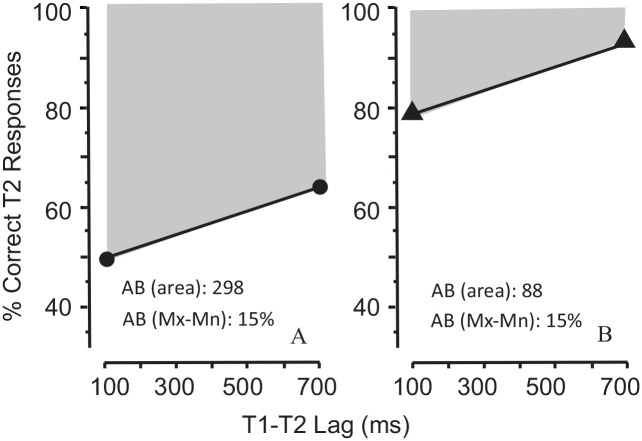
Inconsistency between area and slope measures of the AB. *Note*. In two hypothetical experiments (A and B), the functions of T2 accuracy over lags have the same slope (Mx-Mn, indicating identical AB magnitudes), but different overall levels (causing the area estimates to differ between experiments).

Figures 3A and 3B show the results of two hypothetical experiments that illustrate MacLean and Arnell’s (2012) assertion. In addition, Figure 3 shows why the area (level) method must be regarded as inadequate and potentially problematic. Because the two functions have the same slope, the MAX-MIN method yields identical estimates of AB magnitude (15% in each). But the area method yields a substantially greater AB in Figure 3A (298) than in Figure 3B (88) because Function A is lower than Function B. Clearly, the area method provides an inappropriate estimate of the AB because it is sensitive to the function’s *level*, but not to its *slope* which is the distinguishing characteristic of the AB.

## Does T1 difficulty modulate the magnitude of the AB?

Having reviewed some relevant methodological issues, we now examine the empirical support for the proposition that the difficulty of T1 modulates the magnitude of the AB. Some of the evidence shows unambiguously that T1 difficulty does not modulate the AB (see below). Other evidence is less persuasive because, in the main, it is vitiated by one or both of the following considerations: (a) the estimates of AB magnitude were obtained with the area method, which—as noted above—is a measure of level of T2 performance but not of AB magnitude and (b) the estimates were often constrained by a response ceiling as explained below.

### T1 difficulty and AB magnitude: evidence for independence

**Figure 4.**
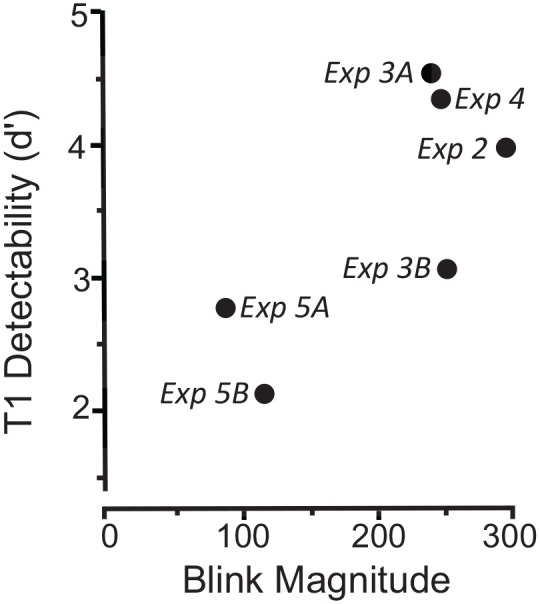
Relationship between T1 detectability and AB magnitude. *Source.* Adapted from Shapiro et al. (1994, Figure 9). *Note*. Lower values of *d*ʹ correspond to harder T1 tasks. On the hypothesis that T1 difficulty is related directly to AB magnitude, the results should lie on an imaginary line from top left to bottom right.

One of the earliest investigations of the relationship between T1 difficulty and AB magnitude was conducted by Shapiro et al. (1994). In a series of seven experiments, T1 difficulty was manipulated in different ways. AB magnitude was quantified as the area bound by the function of T2 correct responses across lags and the 100% ceiling of the response scale. The results are summarised in Figure 4, adapted from Shapiro et al.’s Figure 9 in which T1 difficulty was expressed in terms of *d*ʹ values (the higher the *d*ʹ the easier the T1).

On the hypothesis that T1 difficulty is related directly to AB magnitude, we would expect the values of *dʹ* in Figure 4 to lie along an imaginary line extending from top left to bottom right. But that is not what is seen. Indeed, the pattern of *d*ʹ values is more consistent with the hypothesis that the magnitude of the AB is related *inversely* to T1 difficulty.

Failure of T1 difficulty to modulate AB magnitude has been reported in several other studies. For example, when the magnitude of the AB in Chun and Potter’s (1995, Experiment 4) study is estimated using the slope of the functions (MAX-MIN), there is no evidence that the more difficult T1 produced the larger AB. Indeed, as noted in Figure 2, there was a tendency for the larger AB to be associated with the easier T1. A similar failure has been reported by Seiffert and Di Lollo (1997, Experiment 1). When estimated with the MAX-MIN method over Lags 2–7 (to avoid Lag-1 sparing), there was a tendency for the larger AB to be associated with the easy T1 (14%) than with the hard T1 (9%). Failure of T1 difficulty to modulate the magnitude of the AB has been reported also by Ward et al. (1997) and McLaughlin et al. (2001 Experiment 1) who found that although the difficulty of T1 modulated the accuracy of T1 identification, there was no corresponding modulation of AB magnitude.

### T1 difficulty and AB magnitude: evidence for a positive relationship

Contradicting the above-mentioned outcomes, a number of studies have concluded that T1 difficulty does indeed modulate the magnitude of the AB. The validity of those conclusions is questionable, however, as detailed below. In each of those studies, we estimated the magnitude of the AB from the published graphs using the MAX-MIN method.

In a series of five experiments, Grandison et al. (1997) found that T1 difficulty was related positively to AB magnitude. The correlation between T1 difficulty and AB magnitude was .887. In the study of Shapiro et al. (1994), T1 difficulty was manipulated by the size of the population of letters from which the T1 letter was drawn. In the easy-T1 condition, T1 was one of three letters; in the hard-T1 condition, T1 was one of 25 letters. The results revealed a positive relationship between T1 difficulty and AB magnitude: the AB was 41% when T1 was easy and 50% when it was hard. A similar pattern of results has been reported by Raymond et al. (1995, Experiment 2) who manipulated T1 difficulty by varying the contents of the RSVP frame directly following the T1 frame. The trailing frame contained either an aggregate of random dots (easy T1) or the letter S (hard T1). Again, the results revealed a positive relationship: the AB was larger when T1 was hard (66%) than when it was easy (41%). A positive relationship has also been reported by Raymond et al. (1995, Experiment 3) in which the difficulty of T1 was manipulated by whether the RSVP item following T1 was displaced to a location next to T1 (easy T1) or was not spatially displaced, thus overlapping the T1 item (hard T1). The AB was greater when T1 was hard (64%) than when it was easy (39%). Further evidence of a positive relationship between T1 difficulty and AB magnitude has been reported by Visser (2007, Experiment 2, Figure 3B). T1 was the letter C or G which was displayed either alone (easy T1), or with another letter (medium T1), or with another four letters (hard T1). The AB was smallest (9%) when T1 was easy, it was intermediate (41%) when T1 was of medium difficulty, and largest (53%) when T1 was hard. Homologous positive relationships have been reported in other studies (e.g., Drew et al., 2014; Ouimet & Jolicœur, 2007; Visser & Ohan, 2007).

### Addressing the contradictions

Emerging from the present survey are two classes of experiments. In one class, T1 difficulty was found to modulate AB magnitude; no such relationship was discovered in the other class. It is now appropriate to ask what substantive, procedural, or design characteristics separate the two sets of experiments. On inspection, one such characteristic is readily observable: in all the experiments that found a positive relationship, the functions for the different manipulations of T1 difficulty converged to a common level close to the 100% limit of the response scale. None of the experiments in which T1 difficulty was found not to modulate AB magnitude exhibited that characteristic: all functions were broadly parallel indicating independence (i.e., additivity as distinct from interaction) of T1 difficulty and AB magnitude.

**Figure 5.**
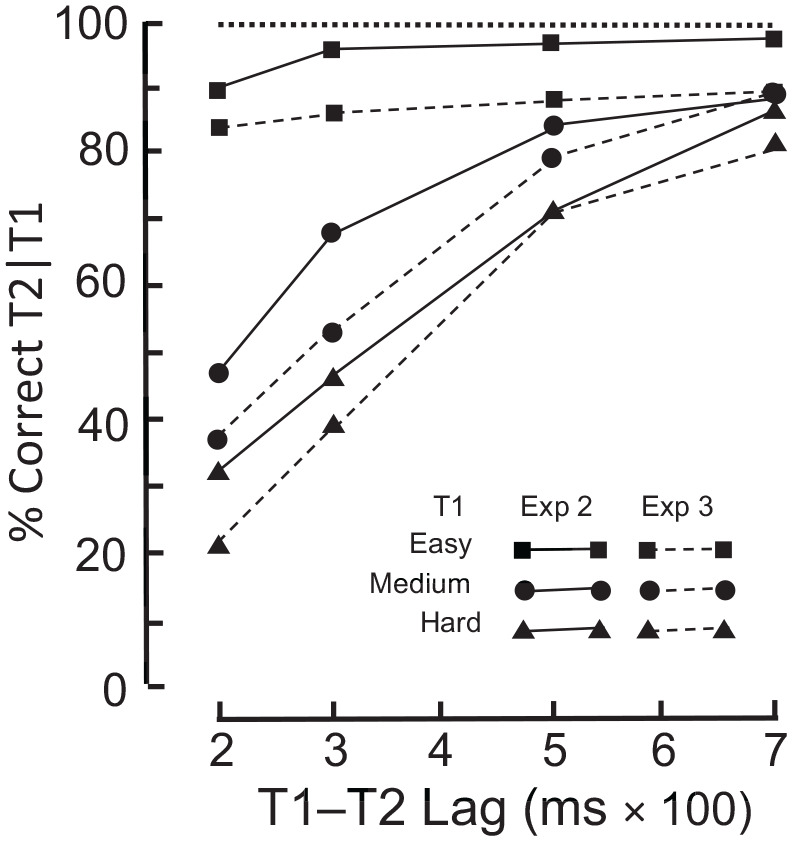
Illustration of performance constraints due to the 100% limit of the response scale. *Source.* Adapted from Figures 3B (Experiment 2) and 4B (Experiment 3) of Visser (2007).

Convergence of the AB functions to a common level strongly suggests the presence of a ceiling that constrains T2 performance to values at or near ceiling. An apposite example of such a constraint comes from the above-mentioned study of Visser (2007, Experiments 2 and 3) in which the difficulty of T1 was either low, medium, or high, as illustrated in Figure 5. It is immediately obvious that all the functions in Figure 5 converge towards a high level of T2 accuracy which is constrained by the 100% limit of the response scale.

**Figure 6.**
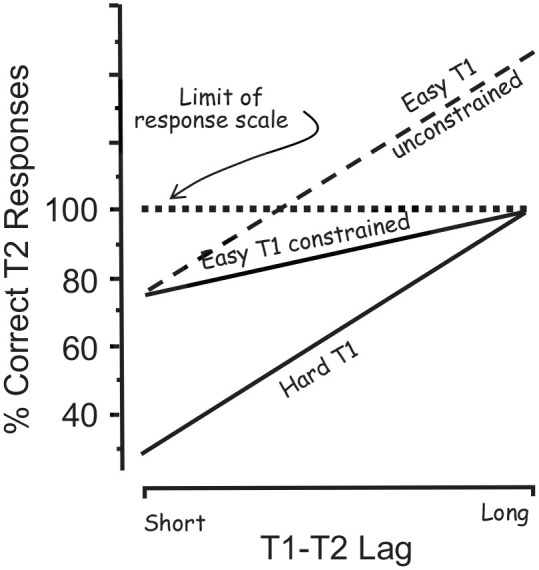
The 100% ceiling can constrain the slopes of the response functions. *Source.* Adapted from MacLean and Arnell (2012, figure in Box 1). *Note*. Illustration of how the 100% limit of the response scale can reduce the slope of the functions of T2 accuracy over lags, thereby causing a corresponding reduction in the estimated magnitude of the AB. Depicted is the case in which the functions for the hard-T1 and the easy-T1 (unconstrained) conditions are parallel, as is the case in experiments discussed in the text. The slope of the function labelled “Easy T1 constrained” is rendered shallower by the ceiling imposed by the limit of the response scale. Comparison of that slope with the slope of the function labelled “Hard T1” would lead to the erroneous conclusion that T1 difficulty modulates the magnitude of the AB.

Pursuing this issue a step further, consider a hypothetical experiment in which a stimulus is displayed for durations of 20, 40, 80, or 160 ms. Suppose that the observer’s accuracy increases with increasing exposure duration, reaches the 100% ceiling at an exposure duration of 80 ms, and remains at ceiling at an exposure duration of 160 ms. Does that mean that the observer lacks the capability of identifying 160-ms stimuli with greater ease than 80-ms stimuli? Or does it mean that the expression of the observer’s true capability is constrained by the response ceiling? If the ceiling were to be removed, the observer’s true capability would come to the fore. These contingencies are illustrated in Figure 6, redrawn from Panel A of the figure in Box 1 of MacLean and Arnell (2012).

It is clear from Figure 6 that when the easy-T1 and the hard-T1 functions converge to a common level—as was the case in the studies that found a positive relationship between T1 difficulty and AB magnitude—the magnitude of the AB for the easy T1 was probably underestimated. In the case of converging functions, the easy-T1 condition would not have produced an actually smaller AB. Rather, the 100% ceiling would have constrained the range of AB values that could be computed, thus producing an underestimate of AB magnitude as illustrated in the “Easy T1 Constrained” function in Figure 6.

Response-scale limitations are not the only source of ceiling constraints. At least, two other forms of constraints have been identified: data limitations and resource limitations (Norman & Bobrow, 1975). Data limitations occur when a target is physically degraded so that its identification is impaired. Resource limitations occur when the target’s processing requirements exceed the observer’s processing capability. There are several ways in which these ceiling problems can be obviated. A direct way of obviating the 100% limit of the accuracy response scale is to use reaction time (RT) as the dependent measure. By its very nature, RT is not constrained by a response ceiling. When using RT, however, appropriate precautions need to be taken to avoid the floor constraints that are sometimes encountered with that dependent measure.

**Figure 7.**
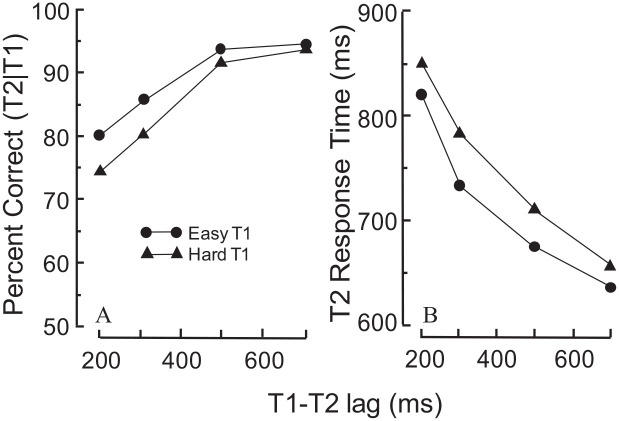
The response functions are ceiling-constrained with accuracy—but not with RT—measures. *Source.* Adapted from Lagroix et al. (2015). *Note*. The stimuli and procedures in Experiments A were exactly the same as in Experiment B, except for the dependent measure which was accuracy in Experiment A and RT in Experiment B. The converging functions in Experiment A create the impression that T1 difficulty modulated the magnitude of the AB. But that conclusion is erroneous because the two functions are constrained by the ceiling imposed by the 100% limit of the response scale (see Figure 6). That is not the case in Experiment B in which the RT measure was free from ceiling constraints. The parallel functions in Experiment B indicate independence of T1 difficulty and AB magnitude.

A direct comparison of accuracy and RT as dependent measures was done by Lagroix et al. (2015) who explored how the estimated relationship between T1 difficulty and AB magnitude is affected by the choice of dependent measure. In each of two experiments, T1 difficulty was manipulated by the congruence (easy T1) or incongruence (hard T1) between the physical size and the numerical value of the T1 stimuli (the numerical Stroop; Szűcs & Soltész, 2007). The two experiments were identical in every respect except for the dependent measure which was accuracy for one experiment and RT for the other. The results, illustrated in Figure 7, are unambiguous. The broadly parallel RT functions in Figure 7B indicate that T1 difficulty did not modulate the magnitude of the AB (an analysis of variance revealed no significant interaction with lag; *F*(3,75) = .496, *p* = .686, MSE = 5375.48). The opposite inference can be drawn from the functions in Figure 7A, which did show a significant interaction (*F*(54) = 4.02, *p* = .012, MSE = 16.42). But that inference would almost certainly be incorrect because the estimated values of the AB were constrained as they approached the 100% limit of the response scale, as illustrated in Figure 6.

The finding that T1 difficulty did not modulate the magnitude of the AB when RT was the dependent measure is attributable to the absence of a response ceiling that constrained the AB estimates when the dependent measure was accuracy. Further evidence consistent with the independence of T1 difficulty and AB magnitude when the dependent measure is RT (i.e., when ceiling constraints are not an issue) has been reported by Jolicœur and Dell’Acqua (1998, Experiments 2 and 3, but see Experiment 1). It should be noted that while RT measures do not suffer from ceiling constraints, they do suffer from floor constraints, as may have been the case for the remaining experiments in the paper of Jolicɶur and Dell’Acqua.

## Interim summary and conclusion

Even a cursory look at the literature reveals a common belief that the difficulty of T1 modulates the magnitude of the AB. Closer examination, however, shows that belief to be questionable. The studies reviewed in the foregoing can be assigned to one of two categories: those that revealed a positive relationship between T1 difficulty and AB magnitude and those that found no such relationship. All the positive-relationship studies share two characteristics: (a) they employed T2 accuracy as the dependent measure and (b) the accuracy over lags functions converged to a common level as they approached the 100% ceiling of the response scale. Our claim is that the ceiling caused the AB magnitude to be underestimated. Importantly, that underestimation was likely to be greater when T1 was easy than when it was hard. This is because the functions started at a higher level when T1 was easy than when it was hard, but converged to the same ceiling-constrained level, thus yielding a shallower slope for the easy-T1 condition. This necessarily produced a smaller AB, whether estimated by the area method or the MAX-MIN method. On this reasoning, those AB estimates must be regarded as spurious, and the reported positive relationship between T1 difficulty and AB magnitude must be regarded as misleading.

It needs to be emphasised that the critical factor was not the use of accuracy as the dependent measure. Rather, it was the fact that the functions started at different levels, but converged to a common level. Indeed, studies that used accuracy as the dependent measure but yielded parallel functions that stopped short of the response ceiling—thereby avoiding response constraints—invariably found no relationship between T1 difficulty and AB magnitude. Consistent with the idea of independence, no relationship was revealed in studies that employed RT as the dependent measure which, by its very nature, is free from ceiling constraints.

Based on the procedural and substantive evidence marshalled in the present review, the conclusion is inescapable that, contrary to common belief, the difficulty of T1 *does not* modulate the magnitude of the AB. We now turn to an assessment of how the independence of T1 difficulty and AB magnitude impacts theoretical accounts of the AB.

## Theoretical considerations

Given the widespread—if incorrect—belief that T1 difficulty modulates AB magnitude, it is not surprising that most theories have incorporated that belief in their tenets, either directly or indirectly. The effect of T1 difficulty on AB magnitude is often said to be mediated by a delay in T2 processing caused by the requirement to process T1. The greater the difficulty of T1, the longer it takes to process it, the longer the unprocessed T2 will remain vulnerable to events that interfere with its identification. Theories differ as to the nature of those events, but the duration of T1 processing is pivotal to most theories. Extant theories can be classified in one of two groups: theories in which T1 difficulty is said to modulate the magnitude of the AB, and theories that postulate no such dependency.

### Theories in which T1 difficulty modulates AB magnitude

By definition, the finding that T1 difficulty does not modulate AB magnitude is problematic for all theories in this group. It should be noted that the critical factor is not T1 difficulty as such, but the duration of T1 processing as manipulated by means of T1 difficulty. Also, the actual mechanism that mediates the relationship between T1 difficulty and AB magnitude need not be the same across theories. Rather, the critical consideration is that the relevant mechanism is operative only while the processing of T1 is under way.

Clearly affected by the finding of independence between T1 difficulty and AB magnitude is a class of theories known as *bottleneck theories* which are predicated on the twin assumptions that processing occurs in sequential stages and that the transfer of information from a lower to a higher stage is not possible while the higher stage is busy. The earliest—and perhaps best known—bottleneck theory is the *two-stage model* (Chun & Potter, 1995) in which the AB is ascribed to a processing bottleneck that is said to occur when T2 arrives at Stage 1 while Stage 2 is busy processing T1. The AB occurs because, while delayed in Stage 1, T2 is vulnerable to decay and to masking by subsequent stimuli. Clearly, the two-stage model accounts for the AB in terms of the period for which T2 remains vulnerable, namely, for the duration of T1 processing which is known to vary directly with T1 difficulty (e.g., McLaughlin et al., 2001, Experiment 2). Given that T1 difficulty does not modulate the magnitude of the AB, bottleneck theories must be regarded as questionable.

Resource-depletion theories are affected in much the same way (e.g., Dux et al., 2008, 2009; Ward et al., 1996). The basic assumption is that the AB occurs because the resources required for processing T2 are preempted by the processing requirements of T1. A corollary of that assumption is that increments in T1 difficulty will result in greater depletion of resources and, therefore, in a correspondingly greater AB. Disconfirmation of that assumption creates a problem for resource-depletion theories.

Interference models (Nieuwenstein et al., 2005; Raymond et al., 1992; Shapiro et al., 1994; Shapiro & Raymond, 1994) also propose a positive relationship between the duration of T1 processing and AB magnitude. Those models postulate that detection of T1 opens an attentional gate that allows post-T1 items to enter a sensory store where they may interfere with T1 identification. When this happens, the gate is locked, thus excluding T2 if it arrives before T1 is fully processed. It follows from these premises that a difficult T1 will take longer to be fully processed than an easy T1. Therefore, the period for which the gate remains locked—hence the magnitude of the AB—is set by the difficulty of T1. On this reasoning, interference models cannot account for the finding that the magnitude of the AB is independent of the duration of T1 processing, as modulated by T1 difficulty.

Like the models mentioned in the foregoing, the temporary loss of control (TLC; Di Lollo et al., 2005) model postulates that the duration of T1 processing—as determined by T1 difficulty—modulates AB magnitude. Specifically, the model proposes that the AB occurs when an input filter tuned to T1 is disrupted by a post-T1 distractor while T1 is being processed. Reinstatement of the filter is delayed until T1 processing is completed. During this delay, T2 is vulnerable to decay and masking, which bring about the AB.

Similar considerations apply to the threaded cognition model of the AB (Taatgen et al., 2009). The detection of a distractor while T1 is being processed elicits a protective attentional mechanism that blocks processing of new stimuli until T1 processing has been completed. Both the threaded cognition and the TLC models are distractor based but, in both cases, the magnitude of the AB is tied to the duration of T1 processing which is held to be determined by T1 difficulty.

Chun and Potter’s two-stage architecture has been incorporated and expanded in the *episodic simultaneous type*, *serial token* (*eSTST*) model of Wyble et al. (2009). In the eSTST model, the AB occurs when the allocation of attention to new items—notably the T2 item—is suppressed while the encoding of T1 is under way. Given that the duration of T1 processing is modulated by T1 difficulty, the eSTST model clearly assumes a positive relationship between T1 difficulty and AB magnitude. That assumption, however, is negated by the empirical evidence.

### Theories in which T1 difficulty is unrelated to AB magnitude

Only two theories can be classified in this category: The *Boost and Bounce* (B&B; Olivers, 2010; Olivers et al., 2007; Olivers & Meeter, 2008) theory and the *locus coeruleus–norepinephrine* (LC–NE; Nieuwenhuis et al., 2005) theory. There is no need for either theory to explain the relationship between T1 difficulty and AB magnitude simply because T1 does not play an active role in either theory.

According to the B&B model, a distractor that occurs directly after T1 triggers a period of suppression that inhibits the processing of trailing items. The AB occurs when one of those trailing items is T2. What distinguishes B&B from other models is the tenet that the AB is time-locked not to T1 but to the distractor directly following T1. This makes T1 difficulty and its relationship to the AB an irrelevant consideration.

Also free from the requirement to explain the relationship between T1 difficulty and AB magnitude is the LC–NE theory of Nieuwenhuis et al. (2005). The onset of T1 is said to trigger a brief period (~200 ms) of phasic activity in LC, followed by a refractory period that can last up to 400–500 ms. Activation of the LC–NE neuromodulatory system causes the secretion of norepinephrine which facilitates the processing of T1. The secretion of norepinephrine is suppressed during the ensuing refractory period, impairing the processing of incoming stimuli. The AB occurs if T2 arrives during the refractory period. Much as in the B&B model, T1 acts simply as a trigger for the eventual refractory period leading to the failure to process T2.

## Concluding comments

Does T1 difficulty modulate AB magnitude? In the course of answering that question, we first examined—and questioned—the dependability of the empirical evidence. We found that, contrary to common belief, the bulk of the evidence indicates that the difficulty of T1 does not modulate the magnitude of the AB. We then reviewed extant theories of the AB and found that only two could account for the independence of T1 difficulty and AB magnitude. This, we hasten to note, does not mean that other theories cannot be revised to accommodate that independence. But, in their current form, those theories must be regarded as wanting.

An important issue regarding the impact of T1 difficulty on T2 performance needs to be raised. The very same evidence that T1 difficulty does not affect the *slope* of the function of T2 accuracy over lags, also shows that T1 difficulty has a marked effect on the overall *level* of T2 performance. Consider, for example, Chun and Potter’s (1995) results illustrated in Figure 2. The two functions are statistically parallel, indicating that T1 difficulty did not modulate the function’s slope (i.e., it did not modulate the magnitude of the AB). In contrast, the two functions differ significantly from one another in overall level. Given that the T2 stimulus was in common, the level differences must be ascribed to differences in T1 difficulty.

Besides the study of Chun and Potter (1995), this pattern of results occurred in studies in which the functions were parallel and stopped short of a response ceiling, whether the dependent measure was accuracy (Seiffert & Di Lollo, 1997) or RT (Jolicœur & Dell’Acqua, 1998; Lagroix et al., 2015). Most other studies also show that T1 difficulty modulates the level of T2 performance (e.g., Grandison et al., 1997; Shapiro et al., 1994; Visser, 2007). Regrettably, an assessment of the independence of level and slope is not possible in those studies because the functions converged towards a response ceiling, thereby confounding the relationship between slope and level, as exemplified in Figure 6.

The pertinent question now becomes: why does T1 difficulty modulate the overall level of T2 performance but not the slope of the T2 function over lags? Obviously, an answer to that question must await further investigations. At this stage, what can be asserted with confidence is that the independence of level and slope points to the operation of separate underlying mechanisms.

Although the precise nature of those mechanisms remains unknown, some speculations may be in order. One such speculation can be based on Visser’s (2010) idea that size of a memory load consisting of items presented at the beginning of every trial—and held concurrently with the AB task—can be regarded as a form of T1-difficulty manipulation. Thus, studies that manipulated the size of memory load may provide some insight into the mechanisms underlying the effect of T1 difficulty. For example, Woodman et al. (2001) manipulated the size (number of items) of the load to be maintained in working memory (WM) while observers performed a visual-search task. They found that load size modulated the overall level, but not the slope of the function relating RT to load size. The differences in overall level, they suggested, might have arisen from some form of “dual task interference that would impair processes that precede or follow the search process (e.g., response selection); this would lead to an increase in the intercept of the search function, but no change in the slope.” (p. 220).

Of interest to the present work, homologous results have been reported with the AB paradigm by Akyürek and Hommel (2005) and by Visser (2010). The common finding was that the size of the memory load modulated the overall level but not the slope of the accuracy function over lags (i.e., AB magnitude). In addition to extending Woodman et al.’s (2001) visual-search experiments to the domain of the AB, Akyürek and Hommel proposed a mechanism for the dependence of overall level of performance on the size of the memory load.

They proposed that increments in memory load would lead to corresponding increments in the amount of inter-item competition among candidate items in WM. Because the memory load remains active for the duration of any given trial, its effect on the T2 task should be in evidence equally at every lag, which is precisely what was found in the studies mentioned above.
